# Decoding the secret of extracellular vesicles in the immune tumor microenvironment of the glioblastoma: on the border of kingdoms

**DOI:** 10.3389/fimmu.2024.1423232

**Published:** 2024-08-29

**Authors:** Bouchra Ghazi, Zakia Harmak, Mounir Rghioui, Abdou-Samad Kone, Adil El Ghanmi, Abdallah Badou

**Affiliations:** ^1^ Immunopathology-Immunotherapy-Immunomonitoring Laboratory, Faculty of Medicine, Mohammed VI University of Sciences and Health, Casablanca, Morocco; ^2^ Mohammed VI International University Hospital, Bouskoura, Morocco; ^3^ Immuno-genetics and Human Pathology Laboratory, Faculty of Medicine and Pharmacy, Hassan II University, Casablanca, Morocco; ^4^ Mohammed VI Center for Research and Innovation, Rabat, Morocco; ^5^ Mohammed VI University of Sciences and Health (UM6SS), Casablanca, Morocco

**Keywords:** extracellular vesicles, glioblastoma, immune responses, immune tumor microenvironment, intratumoral microbiome, tumor progression, immunotherapies

## Abstract

Over the last decades, extracellular vesicles (EVs) have become increasingly popular for their roles in various pathologies, including cancer and neurological and immunological disorders. EVs have been considered for a long time as a means for normal cells to get rid of molecules it no longer needs. It is now well established that EVs play their biological roles also following uptake or by the interaction of EV surface proteins with cellular receptors and membranes. In this review, we summarize the current status of EV production and secretion in glioblastoma, the most aggressive type of glioma associated with high mortality. The main purpose is to shed light on the EVs as a universal mediator of interkingdom and intrakingdom communication in the context of tumor microenvironment heterogeneity. We focus on the immunomodulatory EV functions in glioblastoma-immune cross-talk to enhance immune escape and reprogram tumor-infiltrating immune cells. We critically examine the evidence that GBM-, immune cell-, and microbiome-derived EVs impact local tumor microenvironment and host immune responses, and can enter the circulatory system to disseminate and drive premetastatic niche formation in distant organs. Taking into account the current state of the art in intratumoral microbiome studies, we discuss the emerging role of bacterial EV in glioblastoma and its response to current and future therapies including immunotherapies.

## 1 Introduction

Brain tumors are highly aggressive and rank among the deadliest cancers (1, 2). The most common brain tumor is glioma, which is globally recognized as the most common primary brain tumor in the central nervous system (CNS) and has the greatest prevalence of all brain tumors (approximately 46%) (3). Gliomas are defined as brain tumors of glial origin (4). Depending on both histology and molecular features, gliomas have been divided into six different families in the 2021 5th edition of the WHO Classification of Tumors of the Central Nervous System (1). The first family, adult-type diffuse gliomas, constitute the majority of primary brain tumors [e.g., glioblastoma multiforme (GBM) and isocitrate dehydrogenase (IDH) wild type]. Insights gained from next-generation sequencing and DNA methylation-based profiling have prompted the characterization of the second family, pediatric-type diffuse low-grade gliomas. Under the banner of “pediatric type diffuse low-grade gliomas”, three are new tumors: diffuse astrocytoma; MYB or MYBL1-altered, polymorphous low-grade neuroepithelial tumor of the young (PLNTY); and diffuse low-grade glioma-MAPK altered. The third family, pediatric-type diffuse high-grade gliomas, is expected to behave aggressively. The fourth family, circumscribed astrocytic gliomas, encompasses a group of well-demarcated typically solid astrocytic tumors. Based on the hierarchical clustering analysis of DNA methylation profiles, the fifth family has been newly recognized, glioneuronal and neuronal tumors, which is a diverse group of tumors featuring neuronal differentiation. The sixth and last family is ependymomas, now classified according to a combination of histopathological and molecular features as well as anatomic site (1). According to the classification of the World Health Organization (WHO), glioma can be categorized into grades I–IV. The most aggressive type of glioma is GBM, classified as a grade IV brain tumor. This entity is characterized histopathologically by necrosis and endothelial growth and associated with high mortality (1, 5, 6).

Although there have been improvements in diagnostic, radiotherapy, and chemotherapy options, the prognosis of gliomas is still poor, especially for malignant and invasive gliomas (7, 8). The prognosis of glioma patients varies according to molecular subtype, with IDH-mutated gliomas generally showing a better disease course and distinct ontogeny compared with IDH wild-type gliomas. There is a clear genetic difference between IDH-mutated and wild-type IDH gliomas, and PTEN mutation is a poor prognostic factor for wild-type IDH patients (9–11). In parallel, several potential pathologic characteristics of glioma have been investigated, which include 1p/19q codeletion, IDH, epidermal growth factor receptor (EGFR), p53, PTEN/Akt pathway, Rb, Ras/MAPK pathway, extrachromosomal DNA, MGMT, TERT, and ATRX (5, 10, 12). Genetic aberrations contribute to the specific glioma subtype and a unique metabolic footprint.

On the other hand, metabolic reprogramming can, in fact, act as a driver of cancer genome modification and oncogenic pathways, through epigenetic, transcriptional, and posttranslational modifications (13). Exactly how cells acquire these hallmarks, and how they can be counteracted is the main question at stake for developing efficacious cancer treatments. Currently, the mainstay of glioma treatment is surgical resection, followed by radiotherapy and chemotherapy (5, 14). However, despite advances in diagnosis and treatment, the prognosis for gliomas remains poor, particularly for malignant and invasive gliomas. This limited efficiency may be due to the intratumoral heterogeneity of tumors. Unfortunately, glioma shows a high biological and genetic heterogeneity associated with exceptional aggressiveness. The discovery of molecular heterogeneity between tumors from different patients as well as within tumors from the same patient suggests the complexity of this cancer. Glioma cancer cells exhibit distinct biological hallmarks including extensive pseudopalisading necrosis (a configuration that is relatively unique to malignant gliomas), microvascular proliferation and angiogenesis, cellular heterogeneity, bilateral invasion, altered metabolism, immunosuppressive microenvironment and heterogeneity, and cancer stem-like cells (10–12, 15, 16). In addition, several potential pathologic hallmarks of glioma have been investigated, which include 1p/19q codeletion, IDH, EGFR, p53, PTEN/Akt pathway, Rb, Ras/MAPK pathway, extrachromosomal DNA, MGMT, TERT, and ATRX (5, 10, 12, 16). Genetic aberrations contribute to the specific glioma subtype and a unique metabolic footprint. On the other hand, metabolic reprogramming can, in fact, act as a driver of cancer genome modification and oncogenic pathways, through epigenetic, transcriptional, and posttranslational modifications (13). Exactly how cells acquire these hallmarks, and how they can be counteracted is the main question at stake for developing efficacious cancer treatments. Moreover, the emerging role of the human microbiome in modulating immune responses and tumor progression highlights the importance of addressing the complex interactions between tumor cells and microbiota. Until now, relatively little attention has been paid to the role of the human microbiome in glioma and particularly GBM. Previous studies have not been conclusive regarding the association between the human microbiome and gliomas and continuous research is required to reshape our understanding of the pathogenesis of glioma (17, 18). High secretion of EV is another characteristic of GBM. While most cells secrete EVs, human GBMs secrete EVs at significantly higher levels *in vivo*, approximately 10,000 EVs over a 48-h period per single GBM cell (19). The RNA-encapsulating EVs were first isolated from patient-derived glioma cells, and thereafter, glioma served as a useful model allowing EV release monitoring, cargo profiling, and intercellular communication investigation (19, 20). In this review, we summarize the current state of EVs in GBM and discuss the interkingdom cross-talk, including the communication established between intratumoral microbiome and immune host cells in the tumor microenvironment (TME). EVs provides a new insight into the pathogenesis of GBM. We also highlight the significant role of EVs in tumor progression, escape, and therapeutic response.

## 2 EV biogenesis, release, cargo, and uptake

**Figure 1:**
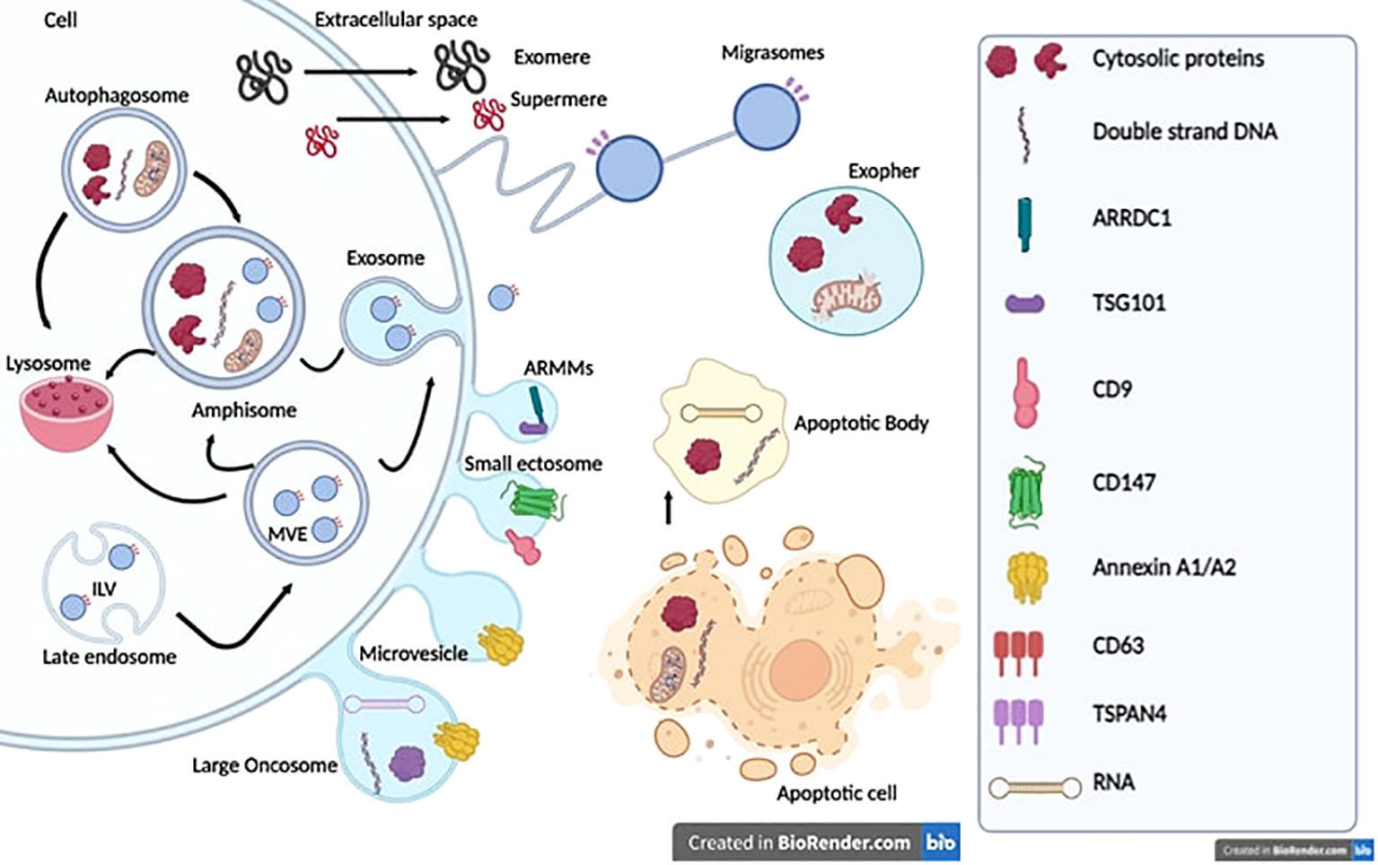
Extracellular vesicle biogenesis and release. Exosomes or small vesicles (30–150 nm) arise from multivesicular endosomes (MVEs) and amphisome. The maturation of the early endosome into the late endosome via inward budding of the endosomal membrane and encapsulation of intraluminal vesicles (ILVs) result in the formation of MVEs. Autophagosomes and MVEs can fuse with amphisomes or lysosomes, thus containing more proteins, nucleic acids, lipids and cytosolic components involved in degradation pathways. Microvesicles or medium/large vesicles (100–1,000 nm) emerge through direct budding of the plasma membrane straight to the extracellular space. Large oncosomes (1–5 μm) arise from tumor cells and contain oncogenic proteins and nucleic acids. ARMMs and small ectosomes (around 150 nm) originate from normal or cancer cells. Exomeres and supermeres (<50 nm) are mostly characterized by specific gene markers, especially TGFBI, ENO1, and GPC1. However, the underlying biogenesis mechanism remains unknown. Migrasomes originate from cell migration and involve structural and adhesion molecules such as Integrins. Exospheres (around 4 μm) result from the release of autophagosomes fused to lysosomes into the extracellular space. Apoptotic bodies originate from cells undergoing apoptosis and contain the remaining components of dead cells, proteins from the nucleus, mitochondria, lipids, and nucleic acids.

EVs are phospholipid bilayer enclosed extracellular spherical structures secreted by cells into the extracellular space (21, 22). Recent advances in isolation and analytical methods have allowed the identification of an ever-increasing number of EV types: microvesicles (MVs), exosomes, apoptotic bodies, small ectosomes, migrasomes, marge oncosomes, and exophers (23–26) (**Figure 1**). EVs have been classified based on their biogenesis, release pathways, size, content, and function (21, 22, 27).

Cells typically communicate with each other by secreting signaling molecules, including proteins, lipids, and nucleic acids. In an effort to maintain homeostasis, influence metabolism, and regulate the immune response, cells can package different signaling molecules in EVs resulting in local and long-distance intercellular communication (21, 28). EVs contain various bioactive molecules both in the lumen and in the surface detected in all tissues and bodily fluids (23–25, 29–32).

**Figure 2:**
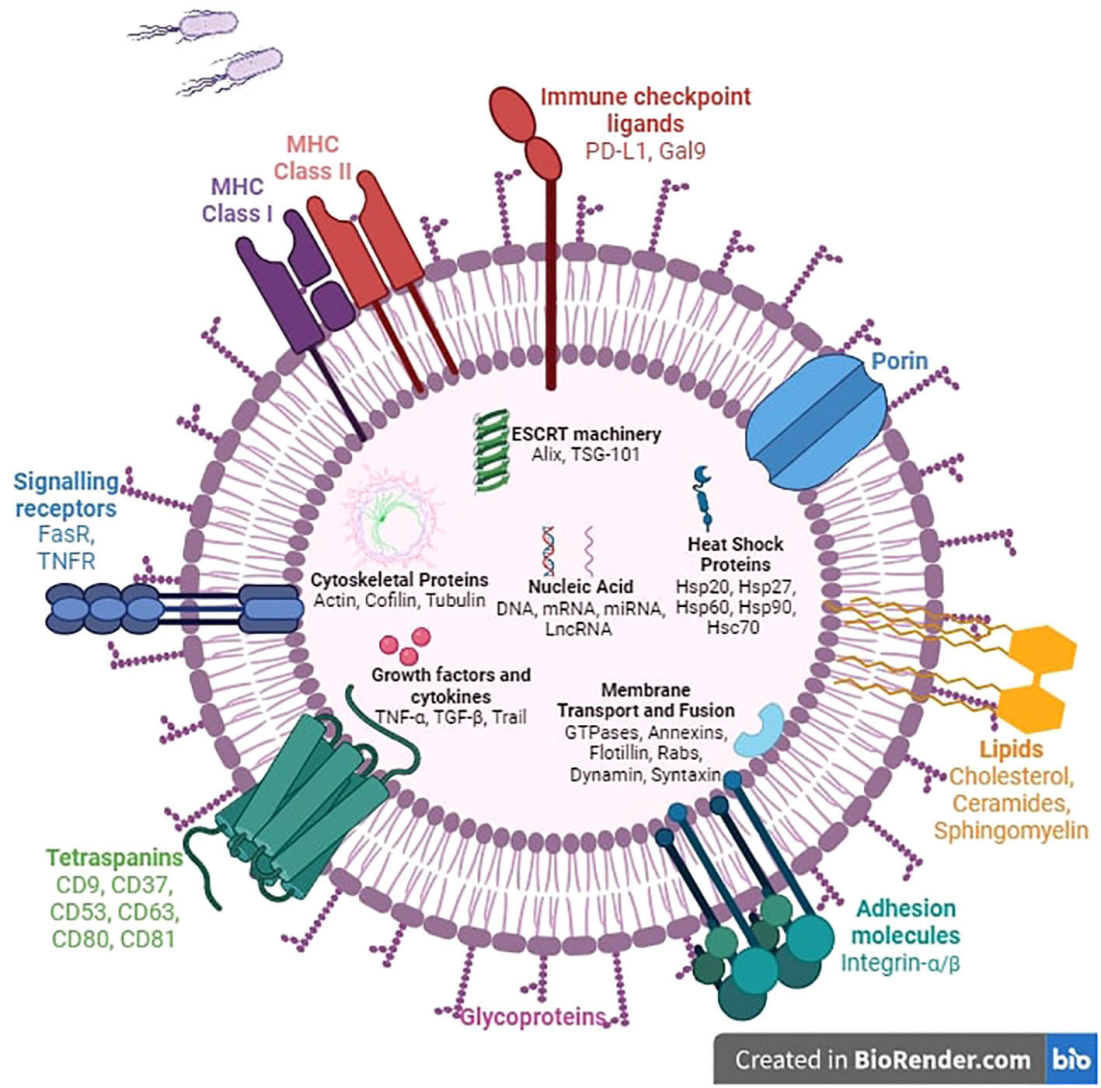
Composition of extracellular vesicles. The structure of extracellular vesicles includes intracellular, transmembrane, and surface components. Most intracellular molecules such as nucleic acids, growth factors, cytokines, and heat shock proteins will be released into the extracellular space and regulate numerous biological mechanisms. The ESCRT machinery, fusion proteins, and membrane transport proteins are involved in membrane remodeling to facilitate exchange between the two sides of the phospholipid bilayer. Surface proteins such as adhesion molecules, immune checkpoint ligands, MHC molecules, and death signaling receptors trigger various mechanisms including cell anchoring, immune cell regulation, and apoptosis.

• EVs have been considered for a long time as a means for normal cells to get rid of molecules it no longer wants, to maintain normal tissue homeostasis, or for cancer cells to promote their malignant tendencies (33, 34). It is now well established that EVs play their biological roles following their uptake by the recipient cell or by the interaction of EV surface proteins with cellular receptors and membranes (19, 26, 32, 35, 36). Indeed, secretion of specific types of EVs has been linked to numerous disease states, including cancer, neurological, and immunological disorders through aberrant signaling (36–39) (**Figure 2**).

Based on their origin, biogenesis, and, thus, their cargo composition, different types of EVs have been classified (21, 22, 27). At least two major modes of biogenesis are known: exosomes or small vesicles (30–150 nm), which starts with the formation of early endosomes that later fuse with the plasma membrane, and MVs or medium/large vesicles (100–1,000 nm) through direct budding of the plasma membrane straight to the extracellular space (**Figure 1**). The process of exosome generation starts with the formation of early endosomes that accumulate intraluminal vesicles (ILVs), by the inward budding of endosomal membranes, during their maturation towards late endosomes or multivesicular bodies (MVBs) (40). Late endosomes and MVBs are a subset of specialized endosomal compartments rich in ILVs, which encapsulate specific sorted proteins and nucleic acids, lipids, and cytosolic components. The fate of MVBs varies according to the proteins that are expressed on their surface. Some function as “delivery trucks” and get transported to plasma membrane via cytoskeletal and microtubule network and undergo exocytosis whereby the ILVs get released as exosomes into the extracellular space (41). Other MVBs function as “garbage trucks” and follow a degradation pathway either by direct fusion with lysosomes or by fusion with autophagosomes followed by lysosomes and thus promote ILV destruction and removal (42).

MVs are a heterogeneous group of membrane‐enclosed vesicles that shed by outward blebbing of the plasma membrane of various cells. These vesicles are loaded with multiple selectively sorted proteins including cytokines, chemokines, proteins involved in cellular signaling and/or migration, lipids, carbohydrates, and genetic material including messenger RNA (mRNA) and microRNAs (miRNAs) (43). Generation of MVs requires membrane lipid and actin cytoskeleton rearrangement to promote plasma membrane budding and subsequent vesicle shedding. The mechanism for classical MV biogenesis, cargo sorting, and shedding is tightly regulated by the small GTP-binding protein ADP ribosylation factor 6 (ARF6). A number of pathways including the small GTPase ARF6/phospholipase D/ERK/myosin light chain kinase pathway mediate phosphorylation of the myosin light chain resulting in actin cytoskeleton contraction at the MV necks in order to enhance myosin contractility and favor the fission and the release of the MVs (44).

Classical MVs are distinguished from the other EVs by size (150–1,000 nm) and lower flotation densities compared with small EVs, and are characterized by the expression of Annexin A1 as a specific protein marker of classical shedding MVs, distinct from both exosomes and arrestin-domain-containing protein 1 (ARRDC1)-mediated MVs (ARMMs) (23, 24, 29). ARMMs are small (inf 150 nm) arrestin domain-containing protein 1 (ARRDC1)-mediated MVs that bud directly from the plasma membrane. The budding of ARMMs requires ARRDC1, which is localized to the cytosolic side of the plasma membrane, and recruits the ESCRT-I complex protein TSG101 to the cell surface to initiate the outward membrane budding (45, 46).

Ectosome cargoes are enriched in cytoskeletal proteins, glycolytic enzymes, and integrins. Initially, they are assembled at the cytosolic face, then differentiated membrane microdomains appear at the cell surface followed by vesicle fission and rapid release to the extracellular space (47, 48). It has been shown that T cells release synaptic ectosomes (~70 nm) at the immunological synapse when they make contact with antigen-presenting cells. Thus, accumulated TCRs on the surface of extracellular MVs bud at the immunological synapse center. This process requires tumor susceptibility gene 101 (TSG101) for sorting of TCRs and inclusion in MVs, and vacuolar protein sorting 4 (VPS4) mediates scission of MVs from the T-cell plasma membrane (49, 50). Furthermore, ectosomes released by platelets induce differentiation of CD4+ T cells into Treg cells and may represent a mechanism of peripheral tolerance (51). Exposure of activated CD4+ T cells to platelet-derived ectosomes decreased their release of IFN-γ, TNFα, and interleukin-6 (IL-6), and increased the production of transforming growth factor-β1 (TGF-β1) (51). Finally, depending again on VPS4, perivascular dendritic cells (DCs) release antigen-bearing ectosomes to share antigen with mast cells and elicit anaphylaxis (52). Thereby, once the IgE-bound mast cells contacted an allergen on the surface of DC-derived ectosomes, they degranulated, releasing their inflammatory mediators (52). This ability of DCs to distribute antigen-bearing ectosomes to immune cells in the perivascular space potentiates inflammatory and rapid immune responses to blood-borne antigens.

Large oncosomes are a class of atypically large 1- to 5-μm MVs carrying abnormal and transforming macromolecules such as oncogenic proteins and nucleic acids (53). They have first been identified in highly migratory and invasive prostate cancer cells and have not been detected in benign tissues (53–56). Their release from tumor cells can be induced by overexpression or constitutive activity of oncoproteins (53, 56–58). Large oncosomes can participate in tumor progression through extracellular matrix degradation and exporting oncogenic content to other tumor or stroma cells, thus reprogramming their phenotype (transcriptomic, metabolism, etc.) and creating a tumor growth-supporting microenvironment (55, 56, 59).

Indeed, other vesicles like apoptotic bodies are formed in a similar way to MVs and considered as an important mediator of extracellular interactions. Apoptotic cell-derived EVs are released from cells entering apoptosis and formed through a process termed apoptotic cell disassembly. They contain the remaining components of dead cells, which include proteins from the nucleus, mitochondria, and plasma membrane; lipids; and nucleic acids like mRNA, long non-coding RNA (lncRNA), ribosomal RNA (rRNA), and miRNA (60–62). Apoptotic EVs have been divided into larger apoptotic bodies (1,000–5,000 nm) and smaller apoptotic vesicles (50–1,000 nm) (63–65). It has been suggested that the molecular cargo of apoptotic EVs differs based on size (66–69). By transporting bioactive molecules, proteins, lipids, and nucleic acids, apoptotic EVs are thought to promote regeneration in skin, bone, and muscle. They also function in inflammation and immune regulation within the TME (61, 70–77).

• Upon secretion into the cellular space, EVs can affect the target cells nearby and further away. Currently, the mechanisms and determinants of EV targeting are not fully elucidated yet (78, 79). There are four major pathways by which EVs can enter a recipient cell: macropinocytosis, lipid raft-mediated uptake, phagocytosis (phagocytosis, micropinocytosis, and lipid raft-, clathrin-, or caveolin-mediated endocytosis), and membrane fusion (26, 78, 80–82). Once the vesicle is internalized, its cargo can be degraded or released into the cytoplasm and transported to the nucleus or the cell membrane. However, it should be noted that the EV functionality does not require internalization, as surface proteins can interact with receptors on the plasma membrane of the recipient cell and initiate intracellular signaling cascades (83). The molecular mechanisms of exosome surface molecules, like tetraspanins, immunoglobulins, proteoglycans, and lectin receptors, binding to target cells are largely unknown (84–86). Exosomal ligands programmed death ligand-1 (PD-L1), TNF, FasL, and TRAIL are interesting potential targets for cancer therapies since their receptors are present on the cancer cell surface (87).

EV uptake and interactions trigger various intracellular signaling pathways that can induce epigenetic modifications in the recipient cells, through transfer of bioactive molecules, and affect cellular behavior and function. Even if EVs are a common communication channel of many cell types in many different contexts and pathologies, the processes involved and the messages they convey are highly personalized. It has been suggested that various aspects of tumor–host interactions are mediated through EVs. From an immunosurveillance point of view, it is becoming increasingly evident that EVs play key roles in cancer progression and drug resistance via promoting cancer-intrinsic pathways as well as immune microenvironment editing toward a pro-tumoral activities (88).

## 3 Pro-tumor roles of GBM-derived EVs in oncogenesis

The RNA-encapsulating EVs were first isolated from patient-derived glioma cells, and thereafter glioma served as a useful model allowing EV release monitoring, cargo profiling, and intercellular communication investigation (19, 20). It has been recognized that functional extracellular RNAs carried by EVs [such as miRNA, mRNA, rRNA, transfer RNA (tRNA), small RNA (sRNA), and lncRNA (89)] play important roles in intercellular communication.

**Figure 3:**
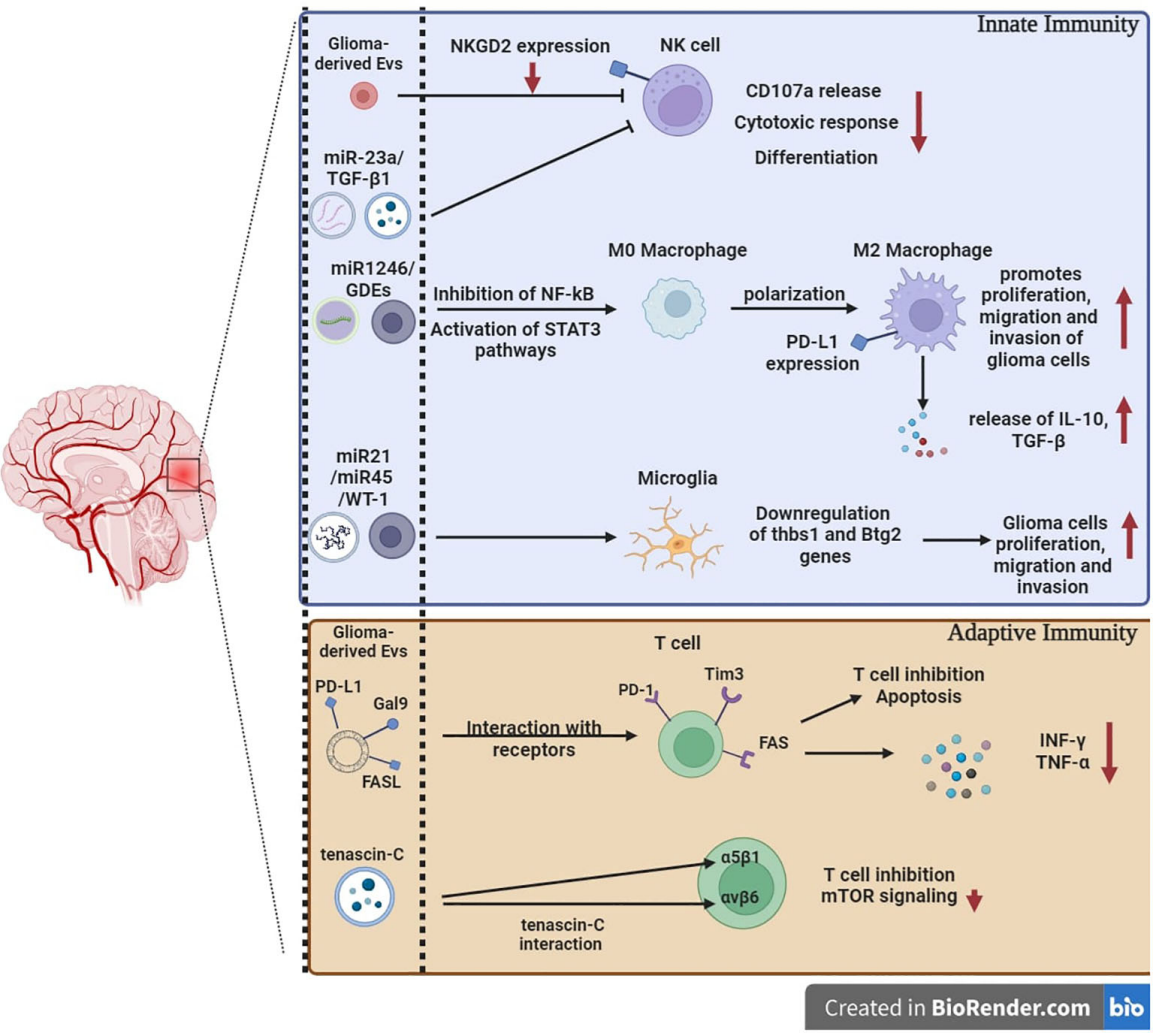
The immunomodulation of glioblastoma-derived extracellular vesicles. GBM-derived EVs include nucleic acids and intracellular and surface proteins. Specific miRNAs and GDEs can either inhibit effector immune cells or promote tumor progression by downregulating co-stimulatory receptors, upregulating co-inhibitory ligands, and activating M2 macrophages that release pro-tumor molecules. GBM-derived EVs expressing co-inhibitory ligands and death signaling ligands can act directly on effector adaptive immune cells and promote apoptosis and T cells’ inhibition. The regulation and differentiation of T cells can be impaired by direct interaction of tenascin-C with integrins involved in T cells’ effector function. miRNAs: MicroRNAs. GDEs: glioma stem-like cell-derived exosomes. Thbs1: thrombospondin 1. Btg2: BTG anti-proliferation factor 2.

While most cells secrete EVs, human GBMs secrete EVs at significantly higher levels *in vivo*, approximately 10,000 EVs over a 48-h period per single GBM cell (19). Furthermore, the presence of MVBs and exosomes inside GBM tissues has been demonstrated by electron microscopy (90). GBM-derived EVs are enriched in a wide variety of signaling molecules, functional RNAs, and lipids that modulate cell–microenvironment communication, support GBM progression, recurrence, and drug resistance through the establishment of a pro-tumoral microenvironment, thereby stimulating GBM cell growth, survival, and invasiveness (91–93). In addition, GBM-derived EVs modulate diverse aspects of the microenvironment like brain endothelial cells reprogramming toward an enhanced and disturbed angiogenesis, altering neighboring normal cells by propagating their oncogenic content, promoting the immunosuppressive properties of microglia, skewing the differentiation of peripheral blood-derived monocytes to activated M2-type macrophages with tumor supportive behavior, and suppressing T cell-mediated immune responses by acting on monocyte maturation and differentiation (94–101) (**Figure 3**).

It has been shown that EVs can serve as a means for short- and long-distance altered receptor transfer in GBM. Chief among specific genetic alterations in GBM is EGFR (102). The estimated rate of EGFR amplification in GBM ranges from 25% to 40%, and many contain the EGFRvIII variant, which is not expressed in normal brain (103–105). GBM-derived EVs containing EGFRvIII can merge with the plasma membranes of cancer cells lacking the receptor and share it with the recipient cells. This event leads to the transfer of oncogenic activity, including activation of transforming signaling pathways (MAPK and Akt), changes in expression of EGFRvIII-regulated genes [VEGF, Bcl-x(L), and p27], morphological transformation, and increase in anchorage-independent growth capacity, resulting in a subsequent transformation of the recipient cells that originally lacked the receptor (91).

It has been demonstrated that hypoxic GBM cells release small EVs (50–200 nm) with pro-angiogenic capacity, due to their enrichment with hypoxia-induced proteins including matrix metalloproteinase-9 (MMP-9), IL-8, platelet-derived growth factors (PDGFs), insulin-like growth factor binding protein (IGFBP)-1 and -3, and caveolin-1, which are associated with poor prognosis in glioma (94). To expand, GBM EVs may also have an immunomodulatory effect, modulating the TME to promote tumor growth via immune escape. In this regard, the cargo of GBM EVs comprised several immunomodulating molecules (i.e., TGF-β, IL-10, and heat shock proteins) as well as PD-L1, which binds to the PD-1 receptor on the surface of tumor-infiltrating lymphocytes (TILs) and leads to cancer immune evasion through the inhibition of T-cell responses and, in turn, decreased survival outcomes in cancer patients (106–108).

It has been suggested that the communication between GBM and surrounding cells in the microenvironment enhances the process of transformation and thereby feed continuously the tumor with newly transformed neoplastic cells. Glioma-derived EVs can be taken up by virtually every cell type in the brain microenvironment, including astrocytes, microglia, and microvascular cells, and therefore skewing their phenotypes toward tumor-promoting cells and thereby supporting the tumor progression or recurrence (19, 96, 109–114). For example, the immunosuppressive properties of microglia can be promoted after taking up EVs from GBM cells, underpinned partially by RNA-mediated mechanisms (98, 99). GBM-derived EVs also enhance the neovascularization capacity of human brain endothelial cells, by reprogramming brain endothelial cells toward highly distinct gene regulatory responses that converge on malignant vasculature, a hallmark of the GBM (95). A variety of mutated or amplified oncoproteins in glioma, such as P53, TERT, and RAS, can transform astrocytes to neoplastic cells *in vitro* and *in vivo*. It has been demonstrated that glioma-derived EVs are efficiently transferred to astrocytes, which provide a niche for glioma-initiating cell production in the brain microenvironment. EVs supports the self-renewal, proliferation, and anchorage-independent growth of human astrocytes, by enhancing aberrant signaling pathways commonly observed in GBM: activated Ras, telomerase, or simultaneously inactivated p53 and pRb pathways (102, 115). Furthermore, GBM EV-treated normal human astrocytes exhibit increased migratory capacity and enhanced cytokine production, which lead to increased tumor cell growth. GBM EV-treated normal human astrocytes also acquire tumor-like signaling pathways and exhibited colony-forming behaviors, suggesting that GBM EVs drive astrocytes to a tumorigenic phenotype that could impact the local environment to benefit the tumor itself (110). The transcriptomic analysis of the recipient astrocytes suggested dynamic changes of metabolic genes upon EV uptake, particularly factors of glycolysis, associated with activation of mitochondrial respiration and glycolysis in these cells (116–118).

Recently, it has been proposed that mRNAs encoding glycolytic enzymes and mitochondrial oxidative phosphorylation (OXPHOS) system factors secreted by glioma cells in EVs reprogram the metabolism of the GBM microenvironment (97). The direct transfer of mRNAs encoding metabolic factors may explain part of the observed metabolic alterations induced in astrocytes (97). Several classes of mRNAs, with the complete open reading frames and protein-coding potential, have been identified to be highly enriched in GBM EVs and have been suggested to exert functional effects in the recipient cells. Notably, transcripts for ribosomal proteins (RPs), mitochondrial OXPHOS system, and glycolytic factors represent the dominant fraction of the GBM EV-mRNA species. Ribosome activity is a critical regulator of growth and metabolism as ribosomal availability affects glycolysis and mitochondrial function (119). For instance, enolase-1 mRNA is encapsulated in both GBM stem cells’ MVs and exosomes. This mRNA encodes alpha-enolase, a key glycolytic enzyme, frequently overexpressed in glioma and multiple other cancers (120). Intriguingly, with its direct role in glycolysis, enolase-1 promotes cell proliferation by regulating the PI3K/AKT signaling pathway and is associated with glioma progression (120).

The EV-mediated transfer of oncogenes may contribute to the dysregulated proliferative and metabolic phenotypes observed in the astrocytes. Zeng and colleagues have shown that c-Myc and CCND3 mRNAs were encapsulated in glioma MVs (97). It is evident that Myc deregulation in cancer is a dramatic event in the cell. The MYC oncogene encodes a transcription factor, c-Myc, which tightly controls metabolic pathways to maintain cellular homeostasis in nontransformed cells. c-Myc is often genetically deregulated in cancer and correlates with the grade of glioma malignancy (121). Deregulated cancer metabolism impacts Myc expression and function. Consistently, it is no longer surprising that Myc operates at the intersection between metabolic pathway activation and gene expression. Furthermore, the uncontrolled growth of gliomas can be driven by frequent mutation and transcriptional dysregulation of cell cycle factors, such as cyclin D3 encoded by CCND3, and involved in the control of G1/S phase transition (122, 123).

To conclude, GBM-derived EVs mirror the molecular features of the tumor and its microenvironment (124–127). The expression level of several GBM-derived EV miRNAs and proteins has been linked to GBM pathogenesis and progression. Direct transfer of these mRNAs and proteins from tumor cells to normal cells within the brain microenvironment may aid/enhance their metabolic reprogramming and drive neoplastic transformation. Transformed cells adapt malignant mechanisms, through protein synthesis and metabolism, to support tumor growth and recurrence via EV-mediated horizontal mRNA transfer.

## 4 Role of EVs in the cross-talk between cancer cells and immune cells

The main infiltrating immune cell populations within the GBM microenvironment are tumor-associated macrophages (TAMs), immunosuppressive myeloid-derived suppressor cells (MDSCs), and CD4+CD25+Foxp3+T-regulatory cells (Tregs) that function as tumor growth promoters and induce T-cell dysfunction (128). However, despite the reduced proportion of GBM-infiltrating T cells, these are among the most critical cells in the antitumor response (129).

### 4.1 Effects on innate immune cells

Innate immune cells present in the GBM microenvironment are represented by cytotoxic NK cells and myeloid cells. It has been shown *in vitro* that GBM-derived EVs can impair the antitumor function of NK cells by suppression of NKG2D activating receptor expression and, thus, NK cell activation (129). It is well known that tumor-derived MVs secreted under hypoxic conditions compromise NK cell cytotoxic responses (130–132). Using multiple tumor models, it has been shown that hypoxic tumor-derived MVs contain two immunosuppressive factors, TGF-β1 and miR-23a, involved in the impairment of NK cell cytotoxicity. Following hypoxic tumor-derived MV uptake by NK cells, the transferred TGF-β1 decreases the NK cell surface expression of the activating receptor NKG2D, thus resulting in NK cell function inhibition. Similarly, miR-23a in hypoxic MVs reinforces the immunosuppression by targeting the expression of CD107a in NK cells (132). On the other hand, activated NK-derived EVs contain the cytotoxic proteins, perforin, granulysin, and granzymes A and B, and are known to induce dose-dependent apoptosis in neuroblastoma by caspase-dependent apoptotic pathways, which is possibly the same mechanism in other tumors like GBM (133).

Growing evidence reveals a central role for myeloid cells in the GBM microenvironment, including DCs, monocytes, macrophages, and microglia, and comprising around one-third of cells of the GBM tumor mass. The proportion of these tumor-associated cells is correlated with the clinical outcome in GBM and other solid cancers (134). Macrophages are of particular interest, as they can acquire different phenotypes according to microenvironment conditions. In solid tumors, including GBM, it is believed that the M1/M2 macrophage paradigm plays a key role in tumor progression. Historically, polarized M1 macrophages are deemed as antitumor cells, because of their enhanced capacity of phagocytosis, cytotoxicity, antigen presentation, and secretion of inflammatory cytokines. On the other hand, the M2-polarized macrophages are commonly regarded as tumor-associated macrophages (TAMs), and associated to pro-tumorigenic outcomes through angiogenic and lymphangiogenic regulation, immune suppression, EV production, hypoxia induction, tumor cell proliferation, and metastasis (135).

In GBM, recent observations suggest that non-polarized M0 macrophages, part of the so-called glioma-associated macrophages (GAMs), are present in the microenvironment (136).

From research within the last few years, released GBM-derived EVs were shown to promote a tumor-supportive macrophage phenotype. *In vitro*, GBM cell line-derived EVs were able to polarize blood-derived monocytes to M2‐like macrophages *in vitro* (99, 100, 137). Moreover, EVs from the hypoxic zones of GBM tumors induce M2 macrophage polarization, *in vitro*, which promote glioma proliferation, migration and invasion. Interestingly, it was shown that a polarization switch towards M2 phenotype exists through EV-mediated delivery of miR1246, which inhibit NF-kB and activate STAT3 pathways in macrophages (138, 139). Furthermore, glioma stem-like cell-derived exosomes (GDEs) are predilected toward monocytes and skew them toward the immunosuppressive M2 phenotype, including PD-L1 expression. GDEs contain members of the signal transducer and activator of transcription 3 (STAT3) pathway that functionally mediate this immune suppressive switch. Mass spectrometry analysis demonstrated that the GDEs are enriched in ephrin and axonal guidance signaling proteins, which are directly transferred to the cytoplasm of the monocytes (140). Glioma-derived exosomes suppress CD3+ and CD4+ T-cell activation and responses by acting on monocyte maturation and formation of monocytic MDSCs rather than on direct interaction with T cells (101). The use of EVs with immune checkpoints is one of the most important mechanisms leading to tumor immune escape and growth in many solid tumors, potentially including GBM. One such mechanism is the receptor Tim-3 that could be engaged by its natural ligand Galectin-9 and lead to immunosuppressive pathways (141). Indeed, it has been shown that cerebrospinal fluid-derived GBM EVs are enriched in Galectin-9 and decrease the antigen-presenting properties of DCs *in vitro*, in a Tim-3-dependent pathway (142).

Glioma-derived EVs exert pro-tumorigenic functions in monocytes and promote their conversion into suppressor cells involved in inhibition of activated CD4+ T cells through upregulation of suppressive cytokines, PD-L1, and lymphocyte antigen six complex (Ly6C), and downregulation of proinflammatory cytokines, MHC II, and costimulatory molecule expression (143).

### 4.2 Effects of EV on T-cell function

In one of the first studies to investigate GBM EVs’ effects on cytotoxic activity of immune cells, it has been reported that mouse GBM EVs promoted tumor growth and inhibited CD8+ T-cell cytolytic activity (144). Interestingly, high and low concentrations of GBM-derived EVs were shown to induce differential modulatory effects on peripheral blood mononuclear cells. Data provided by Hellwinkel et al. revealed that EVs at high concentrations induce selective tolerance associated with decreased IFN-γ secretion and migration capacities in peripheral blood mononuclear cells from healthy donors (145). Accordingly, EV-derived signals can act to suppress different aspects of T-cell responses. Indeed, *in vitro* secreted GSC-derived EVs were shown to be enriched in Tenascin-C that inhibits T-cell proliferation through interaction with α5β1 and αvβ6 integrins on T lymphocytes, associated with reduced mTOR signaling (146). The mTOR signaling pathway plays an essential regulatory role in the differentiation and function of both innate and adaptive immune cells (147). GBM-derived EVs exert an important role in immune evasion through the PD-1/PD-L1 axis (106). The EVs express PD-L1, which binds to PD-1 on activated T cells, resulting in the suppression of T-cell activation and proliferation. This leads to immune escape in glioblastoma patients (148), which confirm the critical function of EVs in facilitating intercellular communication during cancer development (149). Likewise, the expression of PD-L1 on EVs is associated with the mesenchymal GBM subtype and is identified in distinct niches of GBM samples, suggesting a possible involvement in tumor growth (106). Furthermore, treatment of IFN-γ in glioblastoma cells increases expression of PD-L1 and indoleamine 2,3-dioxygenase 1 (IDO1) in EVs, without affecting their size or frequency. IFN-γ-exposed GBM-derived EVs lead to higher differentiation of immunosuppressive MDSCs and NCMs in healthy donor monocytes when compared to naive GBM EVs. Monocytes treated with IFN-γ-exposed GBM EVs exhibit greater suppression of T-cell growth versus those treated with naive GBM EVs. Knocking down PD-L1 and/or IDO1 in GBM cells removes the immunosuppressive effect of IFN-γ-exposed GBM EVs on monocytes, suggesting that these molecules could be considered as possible therapeutic targets to combat GBM EV-mediated immunosuppression (143). It was shown that human GSC-derived EVs inhibited TCR-mediated T-cell activation and proliferation and that these effects arise through direct PD-L1/PD-1 interactions (106). By binding to PD-1 expressed on the surface of activated T cells, PD-L1, expressed by GBM cells and myeloid cells, induce T cell-mediated immune tolerance in tumor local microenvironment, leading to tumor immune escape and tumor growth stimulation (150).

Furthermore, PD-L1 on GBM-derived EVs in combination with other immunosuppressive molecules, FasL, CTLA-4, and CD39, suppresses CD4+ T-cell activation and induces apoptosis in CD8 T cells associated with reduced IFN-γ and TNF-α production, as well as an inhibition of NK cell and CD4+ T-cell response (129). This significant role in immunosuppression can be at least partially mediated by FasL, suggesting that both FasL expressed on GBM cells (by cell–cell contact) and FasL expressed on GBM-derived EVs inhibit T-cell functions (151). In GBM, it seems that the two immunosuppressive mechanisms are involved in T-cell inhibition (1): direct interaction of cancer cell-derived EVs with T cells in the TME, and (2) myeloid cell-dependent T-cell inhibition (101, 152–155). Owing to the natural origin, small size, and short half-life of EVs, monitoring whether *in vitro* results are representative of direct EV-mediated GBM/T-cell interactions *in vivo* remains extremely challenging.

## 5 Emerging role of bacterial extracellular vesicles in cancer

Microbe–host interactions are complex processes that directly and indirectly influence host health by modulating, among other mechanisms, immune responses, metabolism, and integrity of the intestinal barrier (156–162).

Bacteria communicate and interact with nearby bacteria, their environment, and the cells of their host through direct contact and secretion of soluble factors, such as metabolites, lipoglycans, nucleic acids, and proteins (163, 164). Bacteria also communicate via bacterial extracellular vesicles (BEVs), which are likely to be a highly efficient, robust, and economic manner of exchanging information between cells. BEVs are spherical membrane-enveloped particles ranging in size from 20 to 400 nm secreted by both pathogenic and non-pathogenic bacteria. Several lines of evidence show that BEVs can enter the systemic circulation and be detected in human body fluids that disseminate part of the molecular content of the parent bacterium into the extracellular milieu (165, 166). A combination of proteomic and biochemical analyses has demonstrated that BEVs carry a dynamic range of membrane-bound and periplasmic proteins, metabolites, nucleic acids (DNA and RNA), enzymes and toxins, polysaccharides, and peptidoglycan, and their cargo is controlled by specific molecular sorting machineries (165, 167, 168). Accumulating data now indicate that BEVs are heterogeneous in their structure, size, density, molecular cargo composition, and function, with different subtypes that vary based on their different biogenesis routes, the membrane envelope structure, the genetic background of the parental bacterium, and the environmental growth conditions (166). Chromosomal DNA in released BEVs from various Gram-negative pathogenic bacteria like *Pseudomonas aeruginosa*, *Porphyromonas gingivalis*, and *Salmonella typhimurium* is mainly extraluminal with a small fraction in the intraluminal space (169). It has been suggested that external DNA acts in biofilm formation while internal BEV DNA is involved in intercellular cross-talk and horizontal gene transfer of virulence, stress response, antibiotic resistance, and metabolism (169). In addition to innate immune response modulation, pathogenic BEV-derived DNA can be found inserted in the host genome in the nucleus of non-phagocytic cells (e.g., epithelial cells) (169). Like DNA viruses, the possibility that bacterial genetic material could be transferred to human somatic cells and integrated into the host genome is intriguing. The mechanisms underlying these integration events are still poorly characterized. The first proof-of-concept evidence that bacterial DNA sequences integrate into the human genome of cancer cells was reported by Riley and colleagues in gastrointestinal tumors with close proximity to the gut microbiome, suggesting a bacterial DNA role in carcinogenesis (170).

The mechanisms by which bacteria and BEVs impact carcinogenesis as well as tumor progression and therapy response are largely unknown. Several mechanisms have been advanced, including direct tumor-promoting mechanisms such as induction of genomic instability or indirect ones such as generation of proinflammatory and immunosuppressive TME (171, 172). For BEVs, almost all we know about their pathological potential is based on inflammatory disease studies (165, 173). The interkingdom cross-talk, either mutually tumor promoting or tumor inhibiting, between the intestinal, intratumoral microbiome, and host cells in the TME can be mediated through secreted microbial metabolites such as short-chain fatty acids or BEVs. Moreover, it is admitted that gut microbiome-derived BEVs can enter the circulatory system to disseminate to distant organs and tissues and interact with various resident immune cell populations like DCs, neutrophils, and macrophages. The potential role of circulating BEVs have been largely discussed as immunomodulators or even a key driver of premetastatic niche formation in distant organs, a conducive microenvironment to the survival and outgrowth of tumor cells before their arrival at these sites (174). In the same line, a retrospective pan-cancer examination of whole-genome sequencing datasets in the TCGA for microbial reads found unique microbial signatures in tissue and blood that could discriminate between and within most major types of cancer (175). This was confirmed with a study demonstrating that pancreatic adenocarcinoma microbiome composition, which cross-talks to the gut microbiome, influences the host immune response and predicts long- versus short-term survival (176).

The role of BEVs on oncogenesis and tumor progression is likely to be context-dependent. Based on studies of BEVs in infectious diseases, it has been suggested that microbial dysbiosis in cancer could enhance the systemic release of microbiome-derived BEVs, which could promote tumor progression by immunosuppressive reprogramming of the TME. BEVs can drive suppressive cellular monocytic differentiation and indirectly induce T-cell anergy, in a TLR-dependent manner (171). On the other hand, BEVs are able to interact with host cells in distant organs through engaging their microbe-associated molecular pattern (MAMPs) to initiate proinflammatory signaling and drive alterations in the immune landscape, particularly myeloid cells, to foster pre-metastatic niches.

**Figure 4:**
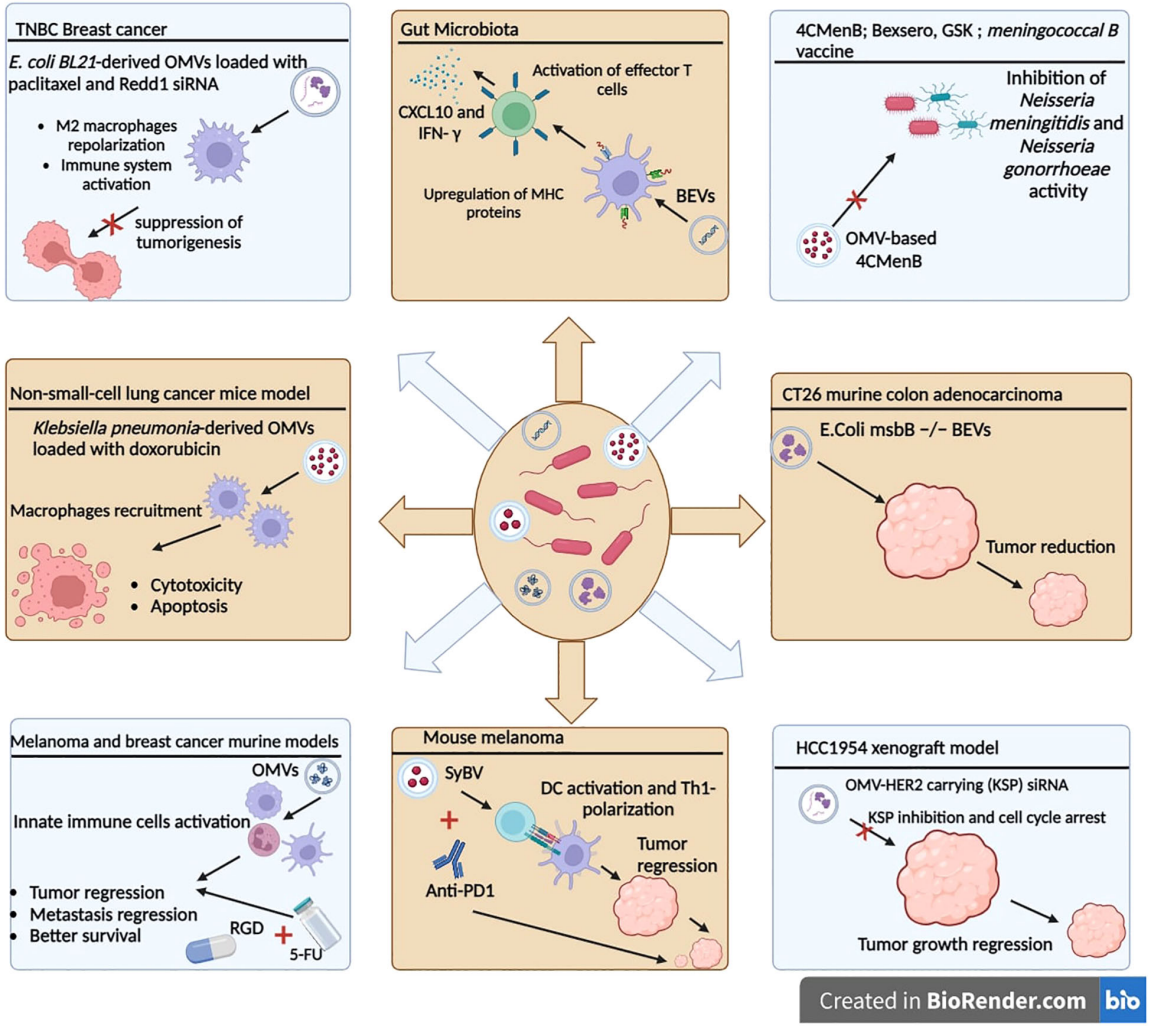
Therapeutic applications of bacterial extracellular vesicles. OMVs can be used in therapeutic strategies and combined with chemotherapy agents or siRNAs to improve the antitumor response. OMVs: outer membrane vesicles. SyBV: synthetic bacterial vesicles. BEVs: bacterial extracellular vesicles.

Gut bacteria-derived BEVs have been shown to prime the host innate immune system with subsequent activation of T-cell responses, in a strain-specific manner. Specific immunomodulatory effects were due, in part, to the differential regulation of miRNAs (177). It has been shown that exosomes released by BEV-activated DCs were enriched in surface proteins involved in antigen presentation and T-cell activation, but differed in the content of immune-related miRNA, depending on the origin of the BEVs (177). From a therapeutic standpoint, to identify new candidates for inclusion in the acellular vaccine formulations, spontaneously released outer membrane vesicles (OMVs) were used as a potential source of key adhesins (178). Adhesins are virulence factors that are surface-bound protein or polysaccharide molecules that confer tissue-specific binding during microbial pathogenesis (179). The clinically approved OMV-based 4CMenB vaccine (4CMenB; Bexsero, GSK) is a four-component protein-based meningococcal B vaccine that was licensed in the European Union in 2013. This vaccine is composed of three highly immunogenic recombinant antigens (factor H–binding protein, Neisseria heparin–binding antigen, and Neisseria adhesin A), as well as OMV containing Porin A subtype P1.4 from the strain NZ98/254 (180). Interestingly, the OMV-based 4CMenB was shown to confer a broad protective antibody response against different *Neisseria meningitidis* and provide a level of cross-protection against *Neisseria gonorrhoeae* because of the molecular similarities shared between the two pathogens (181–183) (**Figure 4**).

As biologically derived entities, the properties of BEVs, like endotoxicity, can be easily determined through molecular biology and genetic engineering approaches. It is expected that, in the future, BEVs will be used as cancer immunotherapeutic agents or cancer vaccines in conjunction with other therapeutic forms, to elicit durable antitumor immune responses. Kim et al. showed the greater immunogenic potential of OMVs over bacteria and the evidence to explore this further in animal models. In a recent report, they showed that systemic intravenous administration of BEVs, from the genetically modified *Escherichia coli* msbB −/− strain (endotoxin-free), in CT26 murine colon adenocarcinoma transplanted mice significantly reduced the tumor volume in a concentration-dependent manner. Biodistribution studies indicated a selective tropism for tumor tissue, which was attributed to the enhanced permeability and retention (EPR) effect given the nano-size range of these OMVs (38.7 ± 4.2 nm) (184). The EPR effect is a property in which the appropriate sizes of nanoparticles leak preferentially into tumor tissue through permeable tumor vessels and are then retained in the tumor bed due to reduced lymphatic drainage (185). The remarkable capability of inducing long-term antitumor immune responses was associated with CXCL10 and interferon-γ cytokine production that can fully eradicate established tumors without notable adverse effects (184). A mutant *E. coli* strain that exhibits less immunogenicity and consequently less toxicity toward human cells was engineered to generate OMVs displaying a cellular selectivity, incorporated genetically with a human epidermal growth factor receptor 2 (HER2)-specific affibody in the membrane as a targeting ligand. The authors used the approach of endogenous loading of antigens to the OMV lumen to generate OMV-HER2 carrying kinesin spindle protein (KSP) siRNA, which is an overexpressed protein in rapidly dividing cancer cells. KSP inhibition cause cell cycle arrest during mitosis by inhibiting KSP function, ultimately leading to cell death. *In vivo* studies in the HCC1954 xenograft model showed that siKSP-packaged OMVs caused targeted gene silencing and induced highly significant tumor growth regression. Interestingly, passive siKSP-loaded OMVs, without HER2 targeting, showed partial tumor regression, offered by the EPR effect, which increases extravasation and retention within the tumor bed (186). Another approach to reduce toxicity and avoid excessive activation of the immune system is the synthetic bacterial vesicles (SyBVs), which are spherical synthetic bacterial vesicles with similar morphology and size to natural bacterial OMVs, but carry less proteins and nucleic acids. The better toxicity profile of SyBVs, compared to OMVs, is due to the limited cytosolic molecular content. Furthermore, SyBVs are capable of engaging cells of the immune system such as DCs and eliciting an adaptive immune response. Co-immunization with SyBV and mouse melanoma derived EVs enhances tumor regression in melanoma-bearing mice in a Th1-dependant manner. Moreover, the immunotherapeutic effect of SyBV was synergistically enhanced by anti-PD-1 inhibitor (187).

Recently, combination therapy using OMVs, as a nanoparticle coating, in tandem with conventional cancer therapies was evaluated. The combination of attenuated *Salmonella*-derived OMVs with chemotherapeutics has been evaluated (188). First, the OMV coating approach has been used to elicit an innate immune response as it travels to the TME, followed by targeting the tumor cells via the arginyl-glycyl-aspartic acid (RGD) peptide on the surface, and subsequently delivering the chemotherapeutic prodrug 5-fluorouracil (5-FU) tegafur (188). The study’s promising results show that successive pretreatment in the mouse model protects against tumor challenge and seems to act like a vaccine. The therapeutic efficacy was also confirmed in melanoma and breast cancer murine models, eliciting repressed tumor growth, reduced metastatic nodules, and better survival than control and tegafur-treated mice groups. In the non-small-cell lung cancer mouse model, apoptotic and cytotoxic effects have been observed with passively loaded doxorubicin in attenuated *Klebsiella* pneumonia-derived OMVs. This antitumor effect of doxorubicin-loaded OMVs was synergized by macrophage recruitment in the TME and enhanced immunogenicity (189). In another study using a triple-negative breast cancer model, genetically engineered *E. coli BL21*-derived OMVs were loaded with paclitaxel and Redd1 siRNA, to enhance immune system activation and chemotherapeutic drug delivery (190). Redd1 is a negative regulator of mTOR signaling and is defined as a key metabolic regulator suppressing tumorigenesis (191). The results show that, after reaching the TME, the paclitaxel is released, followed by OMV-associated siRNA uptake by M2 macrophages leading to tumor-associated macrophage repolarization and tumor immune response activation (190).

Li et al. generated modified OMVs expressing the ectodomain of PD-1 on the surface (OMV-PD-1) capable of inducing a proinflammatory immune response in DCs, and interacting with PD-L1 on the tumor surface (192). Importantly, the engineered OMV-PD-1 can bind to PD-L1 on the tumor cell surface and facilitate its internalization, thereby protecting T cells from the PD-1/PD-L1 immune inhibitory axis. Moreover, in the colon carcinoma cell line CT26 model, 40% of mice exhibited complete tumor regression associated with increased levels of pro-inflammatory cytokines IFN-γ, IL-6, and TNF-α in tumor and serum, and enhanced CD8+ T-cell infiltration. More broadly, the study illustrates the potential of OMVs as a promising agent for cancer immunotherapy capable of regulating the TME and subsequently increasing antitumor therapy efficacy.

Finally, the interaction of OMVs with the host immune system makes them an exciting option for therapeutic cancer vaccines (193–195). Engineered OMVs have elicited an efficient cytotoxic CD8+ T-cell activation by DCs (196). A recent study has demonstrated that OMVs conjugated to antigenic epitope tyrosinase-related protein 2 (TRP2) drive antitumor immunity by eliminating tumor metastasis and inducing a strong cytotoxic T-cell response. These OMVs accumulate in the lymph nodes and carry the potential to efficiently present antigens to the DCs, bringing us one step closer to personalized cancer vaccines (193).

While fecal microbiota transplantation seems to hold promise for many diseases, including cancers, recent events have triggered a greater need to monitor the transfer of antibiotic resistance, which is the main significant risk directly related to fecal microbiota transplantation. Moreover, other causes of deaths following fecal microbiota transplantation have been attributed to heart attack and associated with increased amount of trimethylamine oxide, a metabolite produced by gut bacteria that was shown to be involved in cardiovascular diseases (197). A safer and more controlled way of utilizing the immune-modulatory effect of microbial parts would be to use BEVs. Systemic administration of BEVs directly to tumor-bearing hosts may constitute one of the promising directions of BEVs-based cancer therapy, and could represent a superior alternative to fecal microbiota transplantation (198). The intrinsic properties of BEVs including immunogenicity, a cell-free system, the non-replicative nature, and, thus, safety and nanoscale structure made them become a potential candidate for cancer treatment. Based on their inbuilt adjuvanticity, thermostability, and resistance to low pH and enzymatic degradation, and immunomodulatory properties, several studies tried to evaluate BEVs use for vaccination against infectious pathogens (165, 199).

## 6 EVs in interkingdom communication in the immune tumor microenvironment

The horizontal EV transfer is a new form of intercellular communication that operates at both short and long distances to regulate gene expression, angiogenesis, immune responses, and cell metabolism (112, 200). In cancer, the transfer of EV-associated biomolecules delivers complex biological messages from one cell to another and thereby spread malignant traits across the microenvironment (113). It has been shown in multiple studies that GBM-released EVs are incorporated by neighboring cells in the brain microenvironment, including endothelial cells and microglia, leading to altered phenotypes and functionality, and creating a more supportive TME (95, 98, 109). Bidirectional EV communication shares functional molecules between cancer and stromal cells to facilitate intercellular communication and regulation within the TME.

Recent research has revealed that EVs have a role in the progression of GBM and in the reconstruction of the TME (201) through the interaction with stromal cells, monocytes, macrophages, mast cells, microglia, T cells, astrocytes, and oligodendrocytes (202). GBM-derived EVs also regulate many cellular and extracellular components of the TME, leading to GBM growth and progression (203). GBM-derived EV-mediated interactions may allow TME cells to become activated, notably fibroblasts, microglia, and macrophages. The latter may also adopt either M1 or M2 phenotypes. On the other hand, this cross-talk could potentially result in lineage conversion towards more aggressive phenotypes, such as anaplastic astrocytoma arising from astrocytoma or oligodendroglioma (204). Furthermore, EVs influence other types of cells in the CNS to support the TME. For example, GBM EV-treated astrocytes demonstrate enhanced migration and cytokine production, leading to a tumor-supporting phenotype with a senescence-associated secretory profile (205). Moreover, during treatment with GBM-derived EVs, normal astrocytes demonstrate enhanced migration rates and heightened release of cytokines and growth factors, which could then cooperate with EGF in recruiting precursor cells of mesenchymal origin (110).

Several studies have shown that microglial cells or astrocytes play a critical role in GBM progression (206). Based on recent findings, the complex network of interaction between microglial/astrocytes cells and GBM constitute a potential therapeutic target (207). One reason for this is that because of the glioma-derived EV uptake by astrocytes, the cells possessing high transformation capacity to glioma provide a pool of glioma-initiating cells in the brain microenvironment. It has been shown that EVs enhance self-renewal, proliferation, and anchorage-independent growth properties of human astrocytes, by triggering Ras and telomerase activation or simultaneously p53 and pRb inactivation pathways, the most common signaling aberrations observed in GBM (102, 115). The astrocyte transformation is related to the malignant characteristics of GBM-derived EVs that can elicit additional effects on astrocytes, such as promoting their migration (110).

It has been shown that GBM-derived EVs regulate immune cell activity in the TME (208). Notably, GBM-derived EVs generated upon tumor apoptotic cells may bind to adjacent cells and change their phenotype to become more aggressive. Additionally, these EVs help create an immunosuppressive environment that prevents GBM from antigen-specific detection and death by T cells (208). For example, it has been demonstrated that GBM-derived EVs can facilitate recruitment of Tregs along with additional suppressor cells (209). MDSCs, like other kinds of immune cells, are impacted by GBM and GBM-derived EVs. *In vitro* treatment of PBMCs with GBM-derived EVs raises the MDSC population that exhibit more pronounced immunosuppressive phenotypes and aberrant miRNA profiles approximately 1.5-fold (210, 211). In spite of the increased number and activated state of DCs in GBM patients’ cerebrospinal fluid, the majority of these cells are unable to adequately present tumor antigens. In several situations, GBM-derived EVs severely reduced the antigen-presenting ability of DC as mentioned earlier (142). GBM-derived EV specifically impacts cells of the monocytic lineage, such as monocytes, macrophages, and microglia (100). In fact, GBM-derived EVs can cause peripheral blood monocytes to differentiate into alternatively activated M2-type macrophages. This impact is seen in EVs derived from established cell lines as well as initial cultures of GBM stem-like cells (GSC) (100). Furthermore, GSC-derived EVs influenced primary human microglia, resulting in elevated production of membrane type 1-matrix metalloproteinase, a hallmark for tumor-supportive microglia (100). Moreover, modulatory effects on PBMCs were determined through differential low and high EV concentration effects. These findings suggest that high concentration of EVs can cause specific immune tolerance within the TME (145). Overall, GBM-derived EVs negatively impact the TME immune cells, resulting in an immunosuppression that promotes tumor growth.

Additionally, in glioma, accumulated data suggest that high expression of glycolytic signature genes predicts unfavorable prognosis and immunological heterogeneity (212–217). Interestingly, the GBM-derived EVs can reprogram the metabolism of the recipient pre-transformed astrocytes by activating both glycolysis and OXPHOS, providing a dynamic cross-talk between cancer cells and neighboring cells of the TME. Notably, according to proteomic studies, exosomes contain several key glycolytic enzymes, such as GAPDH, enolase, pyruvate kinase, and phosphoglycerate kinase (108, 218). Several classes of mRNAs are highly enriched in GBM and, most remarkably, transcripts for RP, OXPHOS, and glycolytic factors account for more than 50% of the EV-abundant mRNA (97). In addition to these described means, EV-mediated transfer of oncogenes is another mechanism driving the proliferative and metabolic phenotypes observed in the astrocytes. The full complete coding sequences of c-Myc and CCND3 mRNAs were shown to be encapsulated in glioma-derived EVs (97). The transcription factor c-Myc, which correlates with the grade of glioma malignancy, is known to modulate metabolic reprogramming in the pathogenesis of glioma. c-Myc is also important for the proliferation, growth, and survival of glioma cancer stem cells (121).

It is now well established that the gut microbiome affects the behavior of tumors through blood circulation, bacterial metabolites, and enterohepatic circulation (219–221). Mucosal barriers of the gastrointestinal tract are the hub for interspecies and even interkingdom communication. It is now well established that the gut microbiota is one of the key elements implicated in cancer and shown to modulate anticancer drug efficacy. EVs released by host eukaryotic cells and from prokaryotic symbiotic and/or pathogenic cells, fungi, and parasite-derived EVs meet in intraluminal space and interact constantly with intestinal host cells (222, 223). Interspecies communication between nematodes and host intestinal cells has been recently reported in a mouse model showing that *Heligmosomoides polygyrus* secreted miRNA-loaded EVs suppress host immune response after being internalized by host mice cells (224).

Production of EVs from human parasites, such as trematodes and nematodes, or parasitized cells has been described for a number of parasitic infections (225–227). Recognizing the presence of invading pathogens by germline-encoded pattern recognition receptors is key to mounting an effective innate immune response (228). For example, circulating exosomal miRNAs act as ligands of Toll-like receptors (TLRs) after internalization by target host cells (229). In the same line, mice TLR13 recognize the 23S ribosomal RNA molecule of bacterial pathogen *Staphylococcus aureus* (230). Recently, bacterial DNA integration into the human genome has become a hot topic as it has been found around cancers, such as pancreatic cancer, breast cancer, and colorectal cancer. New lines of evidence support the hypothesis that bacterial integrations and related mutagenesis through lateral gene transfer occur in the human somatic genome and play a role in carcinogenesis (170, 231, 232).

In esophageal squamous cell carcinoma (ESCC), it has been hypothesized that intratumoral microbiota constitute a bridge between digestive tract microbiota and the tumor immune microenvironment, which inevitably influence esophageal carcinogenesis (233, 234). In a study published by Zhang et al., the characterization of the ESCC TME unveils a high abundance of intratumoral *Lactobacillus* and bacterial alpha-diversity, associated with the formation of the immunosuppressive TME depicted by the upregulated PD-L1 expression on epithelial cells and TAMs, and reduced infiltration of NK cells and activated cytotoxic T lymphocytes (235). The authors speculate that intratumoral microbiota might influence patients’ outcomes through the immunosuppressive TME (235, 236).

Moreover, tumor molecular mimicry by gut and extra-gut microbial species producing epitopes that resemble tumor neoantigen epitopes is likely to influence the quality and strength of the immune anticancer response (237). Molecular mimicry occurs when similarities between foreign and self-peptides favor an activation of T or B cells (238). Molecular mimicry can lead to the formation of cross-reactive antigens and/or T-lymphocyte activation and proliferation. Furthermore, epitope spreading, defined as the diversification of epitope specificity from the initial dominant epitope-specific immune response directed against a self or foreign protein, damages healthy tissue and induces apoptosis and concomitant presentation of self- and microbial antigens (239, 240). In an elegant work of Fluckiger et al., the authors reported that MHC-I epitopes derived from a prophage in the gut microbiomes are cross-reactive tumor antigens that enhance immunotherapeutic efficacy in both the preclinical murine model and cancer patients (241). These data highlight the important role of microbiome in modulating antitumor responses and that one of the mechanisms is molecular mimicry.

In CRC, it has been hypothesized that EV-derived proteomes from gastrointestinal tract cancer cells match gut microbiome protein sequences. To investigate this, the CRC EV proteome has been compared with protein sequences from different commensal bacteria and viruses and a number of matching microbial sequences were identified (242–245). Strikingly, the pseudokinase domain sequence in the *B. fragilis* genome matches the PDGFR-α sequence. The oncogenic mutations of PDGFRs and overexpression of PDGF/PDGFRs members are implicated in cancers and are associated with the stage, grade, and poor outcomes of various cancers (246–249). In CRC, it has been suggested that the presence of pseudokinase with activation loop and homology to PDGFR-α in *Bacteroides* spp. may be related to PDGFRα’s role in CRC pathogenesis (250). Hence, matching protein sequences from the host cell-derived EV proteome with the protein sequences of microbiome will help to identify new similarities between bacteria and host cells, including cancer and immune cells and proteins, and understand their functional role in cancer pathogenesis.

Exosomes offer numerous options to study physiological processes and pathologies. Aside from their innate cargo, exosomes from several taxonomic kingdoms have been shown to be loadable with therapeutic agents, acting as nanocarrier for drug delivery (251). Some of the exosomes’ advantages regarding therapeutic purposes are their biocompatibility, stability, low toxicity, penetration into deep tissues, a characteristic zeta potential allowing prolonged circulation, and their intrinsic cell-targeting properties (251–253). Medical potential applications of exosomes include therapeutic approaches such as anticancer therapies, regenerative medicine, microbial vaccines with low immunogenicity that help avoid autoimmunity, cancer vaccines, drug delivery systems, and biomarkers in early diagnosis and therapy monitoring (251).

GBM-derived EVs play vital roles in the induction of the TME, which in the GBM context involves the relationship between GBM tumors and adjacent cells, inducing immunosuppression and stimulating cancer cell proliferation within the brain. Furthermore, in the TME, EVs can serve as a vehicle for both paracrine and endocrine signaling, to adjust metabolic pathways of cells to fit into the objective of the TME.

## 7 Conclusions and future perspectives

To conclude, it is now well established that EV release by cancer cells and other cells within the GBM microenvironment, as well as their presence in biological fluids, is an incontrovertible feature of GBM biology. However, further research efforts are needed to understand and address the functional properties of EVs to potentially gain from the GBM TME-derived EVs.

GBM-derived EVs show strong therapeutic translation potential. Such EVs carry biomolecules that are similar to those that might be secreted by tumor cells, which makes these EVs useful for both diagnostic and therapeutic purposes in GBM. Investigations have shown their ability to cross the blood–brain barrier, allowing imaging agents and treatments to be delivered directly to GBM lesions. Furthermore, EV cargo acts as a pharmacodynamic reporter, providing information about drug distribution and target interaction, which may improve early-phase clinical trials of new therapies. Large amount of data highlights the critical significance of GBM-EVs in improving diagnosis and therapy relating to this challenging brain cancer.

In order to understand the very complex interactions between cancer cells, immune cells, and microbes in the TME, the biologically active concentrations of EVs that actually reach the different intratumoral structures of GBM (e.g., enhancing tumor, necrotic, edema, and non-enhancing tumor) remain to be determined.

Characterizing EV-producing cells provide opportunities to modulate EV biogenesis, release, and cargo content, such as a bioactive proteins or miRNAs. Different strategies can be considered to modulate EV content and biological activities, including biochemical stimuli and genetic modification of the producing cells to overexpress specific proteins or miRNAs.

However, the cargo molecule that will be therapeutically targeted would need to be chosen based on using the relevant non-clinical models for rigorous functional testing of recipient cell responses, ultimately *in vivo*. By gaining a better understanding of the biological functions of EVs and manipulating their biogenesis, research methods in GBM need to be ahead of the game to control their pathophysiological effects in tumor development and produce populations of EVs with antitumor effects.

Finally, isolation and characterization of distinct subsets of EVs from plasma, CSF, or urine have established the proof of principle of using EVs as liquid biopsy biomarkers for the early detection, prognosis, and monitoring of various cancers. Moreover, the non-invasive use of EVs from blood of GBM patients should be envisaged for the monitoring of cancer therapy response efficacy and overcoming cancer drug resistance, notably in immunotherapy clinical trials, with appropriate use of precise and robust EV and cargo characterization.

Finally, further research needs to be performed in this area to comprehensively characterize EV biogenesis and release, and cope with complexity that exists in the interactions between organisms on interspecies and interkingdom levels.

## Glossary

ARF6: ADP ribosylation factor 6
ARMMs: Arrestin-domain-containing protein 1-mediated microvesicles
ARRDC1: Arrestin-domain-containing protein 1
BEVs: Bacterial extracellular vesicles
CNS: Central nervous system
CRC: Colorectal cancer
DCs: Dendritic cells
EGFR: Epidermal growth factor receptor
ESCC: Esophageal squamous cell carcinoma
EVs: Extracellular vesicles
GAMs: Glioma-associated macrophages
GBM: Glioblastoma multiforme
GDEs: Glioma stem-like cell-derived exosomes
HER2: Human epidermal growth factor receptor 2
IDH: Isocitrate dehydrogenase
IGFBP: Insulin-like growth factor binding protein
IL: Interleukin
ILVs: Intraluminal vesicles
KSP: Kinesin spindle protein
lncRNA: Long non-coding RNA
Ly6C: Lymphocyte antigen 6 complex
MAMPs: Microbe-associated molecular pattern
miRNAs: MicroRNAs
MMP-9: Metalloproteinase-9
mRNA: Messenger RNA
MSDC: Myeloid-derived suppressor cells
MVBs: Multivesicular bodies
MVs: Microvesicles
OMV: Outer membrane vesicles
OXPHOS: Oxidative phosphorylation system
PDGFs: Platelet-derived growth factors
PD-L1: Programmed death ligand-1
PLNTY: Polymorphous low-grade neuroepithelial tumor of the young
RGD: Arginyl-glycyl-aspartic acid
RP: Ribosomal protein
rRNA: Ribosomal RNA
sRNA: Small RNA
SyBV: Synthetic bacterial vesicles
TAMs: Tumor-associated macrophages
TGF-b1: Transforming growth factor-b1
TILs: Tumor-infiltrating lymphocytes
TLRs: Toll-like receptors
TME: Tumor microenvironment
Tregs: Regulatory T cells
tRNA: Transfer RNA
TRP2: Tyrosinase-related protein 2
TSG101: Tumor susceptibility gene 101
VPS4: Vacuolar protein sorting 4
WHO: World Health Organization
5-FU: 5-Fluorouracil
